# Electron transfer between complexes III and IV in *S. cerevisiae* mitochondrial membranes

**DOI:** 10.1002/1873-3468.70382

**Published:** 2026-06-16

**Authors:** Ana Paula Lobez, Mikael Oliveberg, Peter Brzezinski

**Affiliations:** ^1^ Department of Biochemistry and Biophysics, The Arrhenius Laboratories for Natural Sciences Stockholm University Sweden

**Keywords:** cytochrome *bc*_1_, cytochrome *c* oxidase, electrochemical gradient, electron transfer, energy conversion, membrane protein, proton transfer, respiratory chain, respiratory supercomplex

## Abstract

Mitochondrial oxidative phosphorylation relies on cytochrome *c* transferring electrons between complexes III and IV. Earlier studies using detergent‐purified complex III‐IV supercomplexes from *S. cerevisiae* showed that this transfer is limited by two‐dimensional cytochrome *c* diffusion. This study investigates this process in membrane‐embedded mitoplasts. The results show that membrane embedment shifts the rate‐limiting step from cytochrome *c*‐mediated electron transfer to the catalytic activity of the supercomplex itself. Up to a cytochrome c : supercomplex ratio of unity, turnover increases sharply regardless of ionic strength. At higher ratios, the rate levels out at 15–20 s^−1^, indicating that the process is no longer limited by salinity‐dependent electron transfer, but rather by the catalytic capacity of complex IV.

## Graphical abstract

Mitochondrial oxidative phosphorylation in *S. cerevisiae* mitoplasts is limited by complex IV catalytic capacity, rather than two‐dimensional cytochrome *c* diffusion. At physiological cytochrome *c* : supercomplex ratios at salinity equivalent to that of 20 mm monovalent salt, activity is maximized, indicating that this low ionic strength accurately mimics physiological electrostatic conditions.

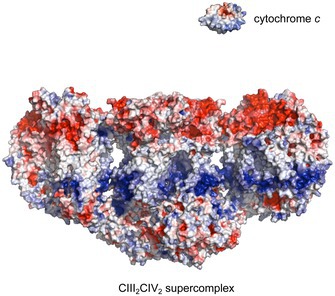

## Abbreviations

**CIII**_**2**_ **/CIV**, complex III_2_/IV

**cyt**, cytochrome

**QH**_**2**_, hydroquinol

Energy conversion in aerobic organisms involves electron transfer through the respiratory chain, which is composed of membrane‐bound respiratory complexes. In the last steps of this process, molecular oxygen is reduced to water. In mitochondria, the O_2_‐reduction reaction is catalyzed by cytochrome *c* oxidase, also referred to as complex IV (CIV) [1]. This enzyme receives electrons from water‐soluble cytochrome (cyt) *c* that is reduced by dimeric cytochrome *bc*_1_ (CIII_2_). Each monomer of CIII_2_ catalyzes oxidation of hydroquinol (QH_2_) to Q and reduction of cyt *c* ^3+^ to cyt *c* ^2+^ (for review, see e.g., [2]). Thus, electron transfer between each CIII_2_ monomer and CIV involves binding of the oxidized cyt *c* to CIII_2_, reduction of cyt *c*, diffusion of the cyt *c* molecule to CIV, docking at the CIV surface, followed by electron transfer from cyt *c* to CIV [3]. In mitochondria, this process takes place in the intermembrane space.

Even though isolation of CIV or CIII_2_ renders functional complexes, results from biochemical and structural studies show that these complexes associate to form supercomplexes [4, 5, 6, 7, 8]. In *Saccharomyces cerevisiae* and *Schizosaccharomyces pombe* yeast mitochondria, CIII_2_ and CIV form supercomplexes composed of CIII_2_ flanked by either one or two CIVs, that is, CIII_2_CIV_1_ or CIII_2_CIV_2_ [4, 9, 10, 11, 12, 13, 14, 15, 16, 17, 18]. In mammalian mitochondria these supercomplexes may also include complex I, forming even larger units of respiratory enzymes referred to as respirasomes [4, 19, 20, 21].

**Fig. 1:**
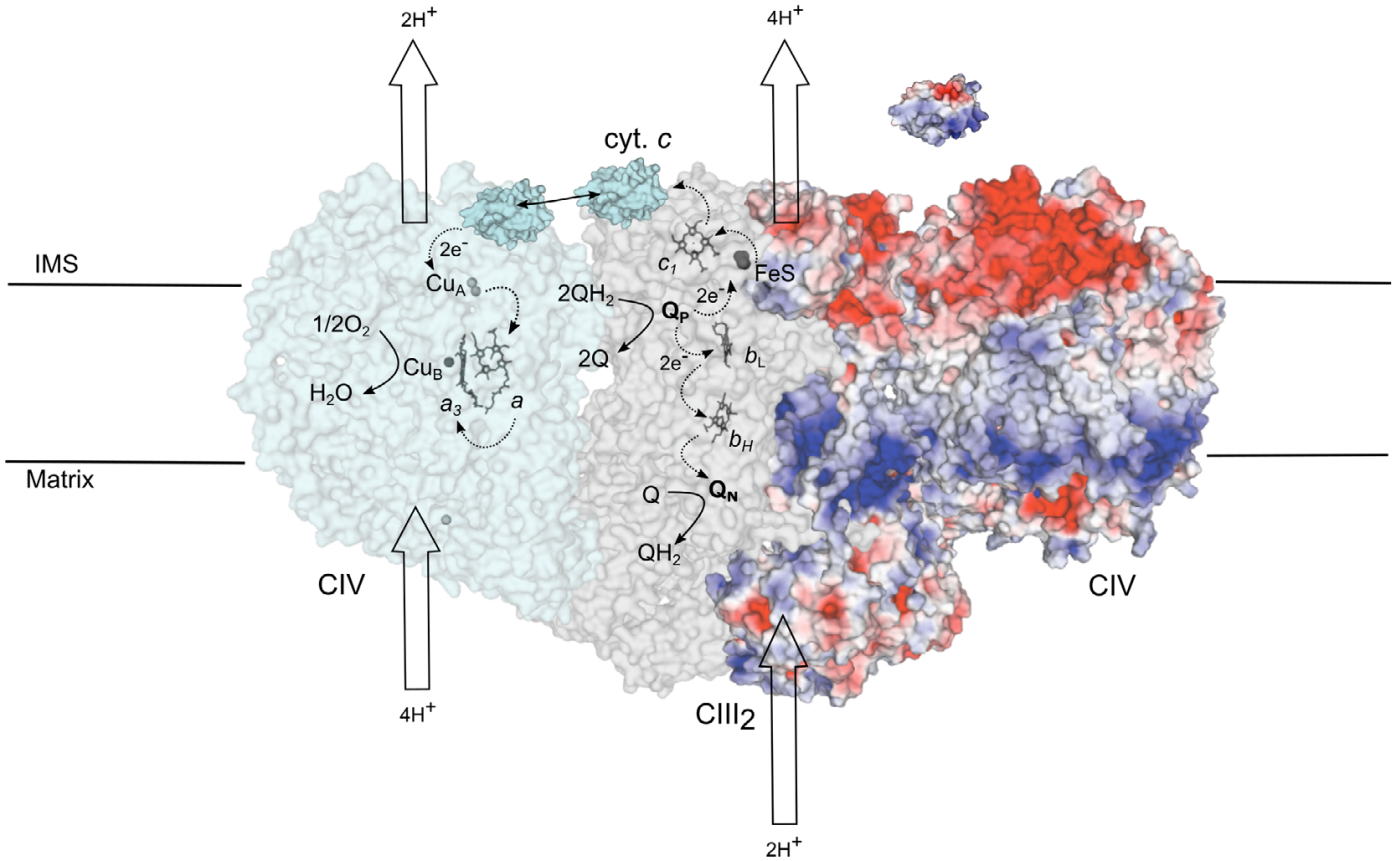
Surface representation of the *S. cerevisiae* CIII_2_CIV_2_ supercomplex and cyt *c*. On the left side, electron transfer between one‐half of the CIII_2_ homodimer and CIV via soluble cyt *c* is shown. Quinol/quinone‐binding sites are indicated as Q_P_ and Q_N_, respectively. Dashed arrows indicate electron transfer, while white block arrows indicate proton‐transfer reactions. On the right side, the electrostatic potential of the supercomplex and cyt *c* is shown. The electrostatic potential from −5 to +5 *k*_B_ *T*/*q*, (*k*_B_ is the Boltzmann constant, *T* is the absolute temperature, and *q* is the unit charge) is indicated from red to blue. The figure was prepared using the standard settings of the APBS tool of the pymol software [57]. The PDB structures for the *S. cerevisiae* supercomplex and cyt *c* are PDBs 6HU9 and 1YCC, respectively.

Cyt *c* is an electrostatically dipolar protein with a positively charged docking surface that surrounds the heme redox cofactor [22, 23]. Surfaces of CIII_2_ and CIV that are exposed to the intermembrane space both carry a strong negative charge, which allows binding of cyt *c* by electrostatic interactions (Fig. 1). When CIII_2_ and CIV are spatially separated, the cyt *c*‐mediated electron transfer is restricted to (three‐dimensional, 3D) through‐solvent diffusion. Association of CIII_2_ and CIV to form supercomplexes opens up a possibility to transfer electrons via 2D diffusion of cyt *c* across the surface of the supercomplex [17, 18, 24, 25, 26, 27, 28]. The latter 2D diffusion process resembles electron transfer via a di‐heme cyt *cc* domain in the obligate CIII_2_CIV_2_ supercomplexes from *Corynebacterium glutamicum* [29, 30] and *Mycobacterium smegmatis* [31, 32, 33].

Intracellular electrostatic conditions are typically mimicked by solutions with an overall salinity equivalent to that of approximately 150 mm monovalent salt [34]. This commonly used standard originates from studies of the intracellular osmotic pressure of, for example, *Escherichia coli* cells, which is comparable to that of a 150 mm monovalent salt solution [35, 36]. As noted by Wennerström *et al*., [36], however, the intracellular electrostatic environment is complicated by the presence of high concentrations of colloidal‐sized macromolecules such as membranes, nucleic acids and proteins that all carry a net‐negative charge [36, 37, 38, 39]. Moreover, charged organic compounds such as ADP/ATP, glutamate or acetate are typically anions. Consequently, electroneutrality in the intracellular medium is maintained by keeping the concentration of small anions (e.g., Cl^−^) at a considerably lower concentration than that of cations such as K^+^ [35, 36]. Therefore, intracellular electrostatic interactions between macromolecules are stronger than those in a 150 mm monovalent salt solution. Wennerström and colleagues [36] estimated that a realistic mimic of the intracellular electrostatic conditions in *E. coli* is equivalent to ~ 20 mm monovalent salt, which results in an increase of the Debye screening length from 0.8 Å (150 mm monovalent salt) to 2.2 Å (20 mm monovalent salt).

Earlier data obtained at 150 mm ionic strength show that one cyt *c* molecule associates with the *S. cerevisiae* CIII_2_CIV_1/2_ supercomplex [24]. In contrast, a recent combined kinetic and cryoEM study showed that at 20 mm salinity, multiple cyt *c* molecules bind to facilitate electron transfer along the supercomplex surface within the CIII_2_CIV_1/2_ supercomplex [28]. In other words, the electron‐transfer events respond critically to the strength of electrostatic interactions [28]. These studies were performed with detergent‐purified supercomplexes where the rate‐determining step of the QH_2_ oxidation : O_2_ reduction activity is the cyt *c*‐mediated electron transfer from CIII_2_ to CIV [3, 24]. In the native setting, however, the supercomplex is surrounded by negatively charged membrane surfaces that effectively compete for cyt *c* binding [40]. In addition, the membrane environment allows a steroid binding site near a proton pathway of CIV to be occupied by a membrane‐derived steroid moiety [41, 42, 43, 44, 45], which has been shown to impair the turnover activity of CIV by slowing proton uptake to the catalytic site [46]. Hence, in addition to modulating the cyt *c*‐binding environment, the presence of the membrane may also act to shift the rate‐limiting step of the supercomplex activity to become limited by the turnover activity of one of its enzyme components.

To address the mechanism of electron transfer within the near‐native setting of the *S. cerevisiae* CIII_2_CIV_1/2_ supercomplex and the effect of the electrostatic environment, we isolated *S. cerevisiae* mitoplasts by removing the outer mitochondrial membrane and investigated the kinetics of electron transfer from QH_2_ to O_2_, mediated by cyt *c* electron transfer from CIII_2_ to CIV at 20 mm and 150 mm KCl. The results indicate that the supercomplex‐cyt *c* electrostatic interactions outcompete any cyt *c*‐membrane interactions to recruit cyt *c* at the supercomplex surface at both 20 and 150 mm monovalent salt. Furthermore, the data show that at cyt *c*:supercomplex ratios larger than ~ 2, in the membrane setting, the supercomplex activity is independent of ionic strength, indicating a rate‐limiting step that is independent of cyt *c* binding and electron shuttling.

## Materials and methods

### Strain and cell growth conditions

*S. cerevisiae* strain BY4741 with a FLAG tag on CIV subunit Cox6 was used [47]. Yeast cultures were grown in YPG media (1% yeast extract, 2% peptone and 2% glycerol) at 30 °C, 180 rpm (Shaker Innova 44R).

### Sample preparation

Mitoplasts were prepared essentially as described previously [48, 49]. Cells were harvested at 6500× ***g*** (5 min, 4 °C) by centrifugation and were washed with 10 mm EDTA buffer at 3000× ***g*** (10 min at room temperature (RT)). The pellet was resuspended in buffer (100 mm Tris and 10 mm Dithiothreitol (DTT) at 30 °C) and incubated while gently shaking (15 min at 30 °C).

After incubation, cells were centrifuged at 3000× ***g*** (5 min, RT), washed with 1.2 m sorbitol, 20 mm KH_2_PO_4_ (pH 7.4) buffer and centrifuged again at 3000× ***g*** (5 min, RT). The pellet was resuspended in a buffer containing 1.2 m sorbitol, 20 mm KH_2_PO_4_ and Zymolyase 20T (3 mg·g^−1^ of solid weight of yeast). For some samples, this buffer was supplemented with 0.3% DTT (subsequent measurements gave similar results in both cases). Cells were then incubated during gentle shaking for 45 min at 30 °C and then centrifuged at 2500× ***g*** (5 min, 4 °C).

Pellet spheroplasts were resuspended in a buffer composed of 10 mm Tris (pH 7.4), 1 mm EDTA, 0.6 m sorbitol and 1 mm phenylmethylsulfonyl fluoride (PMSF), broken using a Teflon homogenizer and centrifuged at 2500× ***g*** (5 min, 4 °C). This step was repeated several times. For some samples this buffer was supplemented with 10 mm Imidazole [49] (subsequent measurements gave similar results in both cases).

The collected supernatant was then centrifuged at 12 000× ***g*** (20 min, 4 °C) two times and the pelleted mitochondria were resuspended in a buffer composed of 0.6 m sorbitol, 20 mm HEPES at pH 7.4. The protein concentration was adjusted to 10 mg·mL^−1^.

Intact mitochondria were centrifuged at 12 000× ***g*** (10 min, 4 °C), washed three times with a hypotonic buffer (60 mm sorbitol, 20 mm HEPES, pH 7.5) to remove the outer mitochondrial membrane and centrifuged at 12 000× ***g*** (10 min, 4 °C). The obtained pellet, containing the mitoplasts, was washed in hypotonic buffer, supplemented with 0.25 m KCl to remove soluble cyt *c* and centrifuged at 12 000× ***g*** (10 min, 4 °C). Mitoplasts were washed again with the hypotonic buffer several times and resuspended to a final volume adjusted to a final concentration equivalent to 0.2–4 μm CIII_2_CIV_1/2_ supercomplex. The isolated mitoplasts were frozen in liquid nitrogen and stored at −80 °C until use.

### UV–visible difference spectroscopy

Optical absorption spectra were recorded using a Cary 100 UV–VIS spectrophotometer (Agilent Technologies, Solna, Sweden) to estimate concentrations of CIII_2_ and CIV in the samples. A reduced‐minus‐oxidized difference spectrum was obtained using a small aliquot of sodium dithionite as a reducing agent. Peaks were fitted for the different heme groups, heme *c*_1_ (554 nm), hemes *b*_H_ and *b*_L_ (562 nm), and hemes *a* and *a*_3_ (603 nm), and their concentrations were calculated using the following difference absorption coefficients: Δε_554_ = 21 mm ^−1^·cm^−1^, Δε_562_ = 51.2 mm ^−1^·cm^−1^, and Δε_603_ = 24 mm ^−1^·cm^−1^ [50, 51].

The orientation of supercomplexes in the mitoplast membranes was estimated as described in the Results section. Conditions were as follows: 1 μm supercomplex, 500 nm cyt *c* from *S. cerevisiae*, 1 mm KCN, 10 mm ascorbate, 100 μm N, N, N′, N′‐tetramethyl‐*p*‐phenylenediamine (TMPD) in buffer containing 20 mm HEPES at pH 7.5, 60 mm sorbitol and 0.1 m EDTA.

### Activity measurements

Decylubiquinone (Sigma‐Aldrich, Solna, Sweden) was dissolved in 99.8% ethanol at a final concentration of 20 mm. Crystals of sodium borohydride (NaBH_4_; Sigma‐Aldrich) were added and the sample was incubated on ice until it turned colorless, indicating the formation of reduced DQH_2_. When the solution was clear, 10–20 μL of 5 m HCl (depending on the amount of NaBH_4_ used for reduction) was added. The sample was then centrifuged for 10 min at 10 000× ***g***, and the supernatant containing DQH_2_ was collected.

The QH_2_ oxidation: O_2_ reduction activity of *S. cerevisiae* CIII_2_CIV_1/2_ supercomplex in mitoplast was measured by monitoring the oxygen‐reduction rate with a Clark‐type oxygen electrode (Oxygraph; Hansatech, King’s Lynn, Norfolk, JL, UK), operated at 25 °C, upon addition of 100 μm DQH_2_. Measurements were done at a concentration equivalent to 20 nm CIII_2_CIV_1/2_ supercomplex and in the presence of variable concentrations of cyt *c* isolated from *S. cerevisiae* (Sigma Aldrich) in buffer containing 20 mm HEPES at pH 7.5, 60 mm sorbitol and 0.1 m EDTA, supplemented either with 20 or 150 mm KCl. The O_2_ reduction rate was negligible in the absence of added cyt *c*. The activity was the same in the absence and presence of the proton ionophore FCCP at 1 μm, that is, the respiratory‐control ratio was one.

The activity of CIV was measured using a Clark‐type oxygen electrode (Oxygraph; Hansatech), operated at 25 °C, to monitor the oxygen‐reduction rate. A baseline was first recorded with 50 μm cyt *c* from *S. cerevisiae*, 10 mm ascorbate, 100 μm N, N, N′, N′‐tetramethyl‐*p*‐phenylenediamine (TMPD) in buffer containing 20 mm HEPES at pH 7.5, 60 mm sorbitol and 0.1 m EDTA, supplemented either with 20 or 150 mm KCl. The reaction was initiated upon addition of mitoplasts at a concentration equivalent to 20 nm CIII_2_CIV_1/2_ supercomplex.

The activity of CIII was measured by following in time absorbance changes at 550 nm in the presence of 1 mm KCN to block oxidation of cyt *c* by CIV. The baseline absorbance was recorded using 50 μm oxidized cyt *c* from *S. cerevisiae* and 1 mm KCN in 20 mm HEPES at pH 7.5, 60 mm sorbitol and 0.1 m EDTA buffer, supplemented either with 20 or 150 mm KCl. The reaction was initiated by adding 100 μm DQH_2_ and then mitoplasts at a concentration equivalent to 20 nm CIII_2_CIV_1/2_ supercomplex.

## Results

**Fig. 2:**
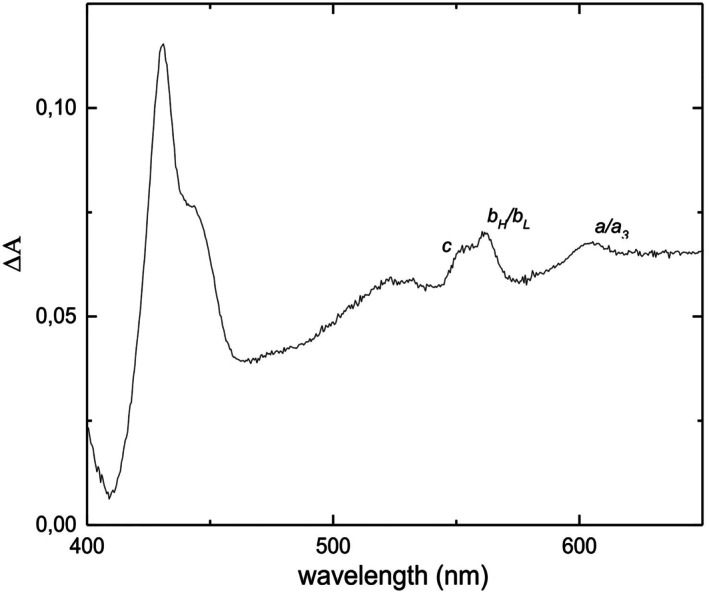
Reduced‐minus‐oxidized difference spectrum. The sample contained isolated mitochondrial inner membranes (mitoplast) from *S. cerevisiae*, which harbor CIII_2_CIV_1/2_ supercomplexes. The difference absorption coefficients used to calculate the concentration of the respiratory complexes were: ε_554_ = 21 mm ^−1^·cm^−1^ (cyt. *c* and *c*_1_), ε_562_ = 51.2 mm ^−1^·cm^−1^ (hemes *b*) and ε_603_ = 25 mm ^−1^·cm^−1^ (hemes *a* and *a*_3_) [50, 51].

We isolated mitochondrial inner membranes (mitoplasts) from *S. cerevisiae*, which contain the CIII_2_CIV_1/2_ supercomplex. The sample was washed to remove any excess cyt *c*. Absorption spectroscopy of the sample exhibited difference spectra consistent with the presence of hemes *a*, *b* and *c* (cyt *c*_1_ of the CIII_2_), without any excess soluble cyt *c* (Fig. 2). The mitoplast preparation used in these studies displayed a respiratory‐control ratio of one, which assures that the data are not influenced by formation of a transmembrane electrochemical gradient.

The supercomplex orientation in the mitoplast membrane and access for cyt *c* were determined by measuring the reduced‐minus‐oxidized difference spectrum upon addition of ascorbate and cyt *c*, and then dithionite. In the former case only the supercomplex population with the cyt *c* binding sites facing the outside is reduced while in the latter case the entire population is reduced. In addition, in the former case only mitoplasts without an outer membrane are reduced. Using this approach, we estimated the fraction supercomplexes that are accessible for cyt *c* binding to be 60%. The fraction supercomplexes reduced by cyt *c* in this experiment reflects the population that is involved in the experiments performed in this study. Therefore, the supercomplex concentration was multiplied by 0.6 in calculations of the turnover activity. The remaining population supercomplexes do not contribute.

The QH_2_ oxidation : O_2_ reduction activity of the CIII_2_CIV_1/2_ supercomplex in mitoplasts was monitored by measuring the O_2_‐reduction rate upon addition of reduced hydroquinol (QH_2_). The experiments were carried out in the presence of variable concentrations of cyt *c* and a membrane sample concentration equivalent to 20 nm CIII_2_CIV_1/2_ supercomplex, at ionic strengths of either 20 mm or 150 mm KCl. Because 90% of CIV in the native *S. cerevisiae* mitochondrial membrane is part of supercomplexes [52], measurement of the O_2_‐reduction rate approximately reflects that of CIV in supercomplex with CIII_2_.

**Fig. 3:**
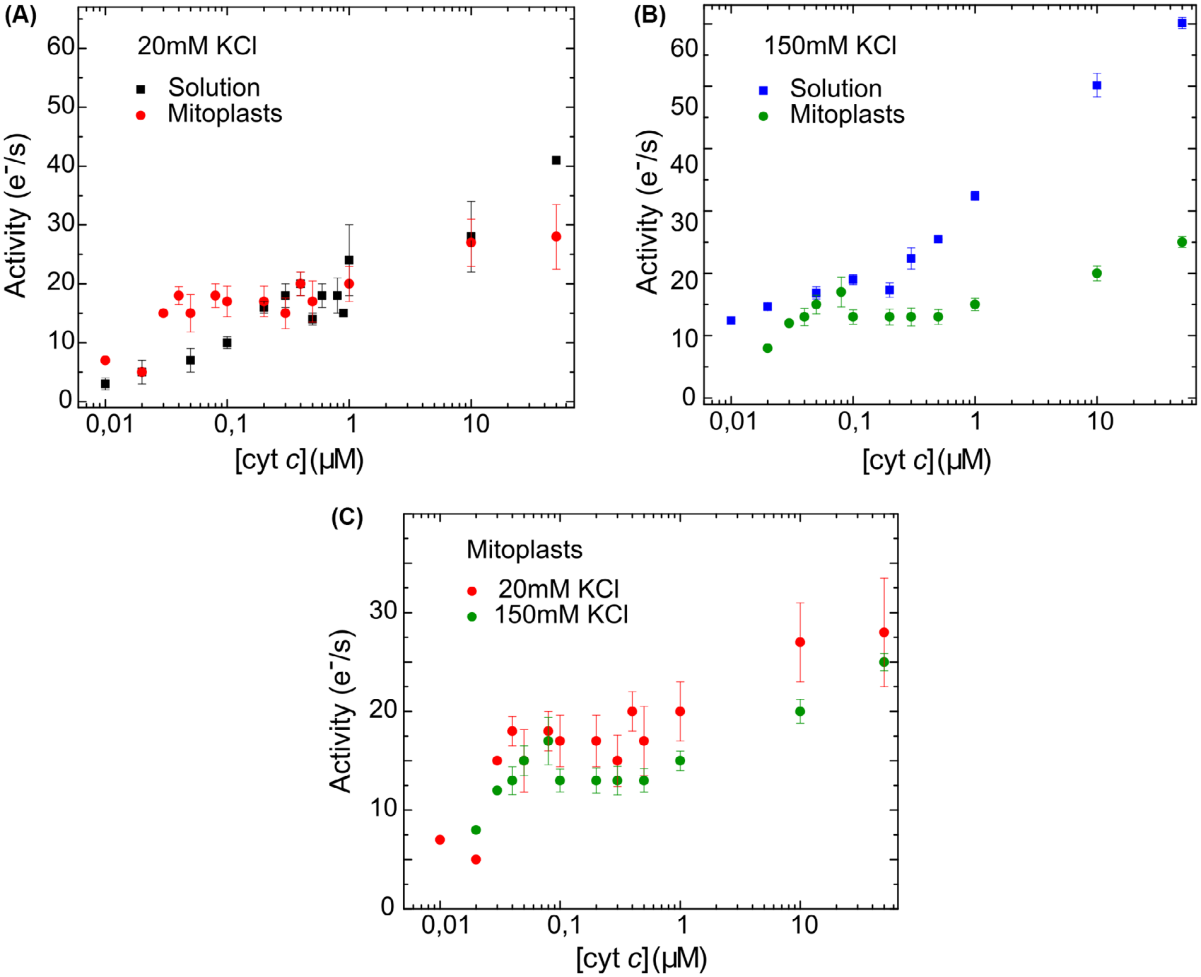
Supercomplex activity as a function of cyt. *c* concentration. QH_2_ oxidation : O_2_ reduction activity of the *S. cerevisiae* CIII_2_CIV_1/2_ supercomplex in mitochondrial inner membranes (mitoplasts), compared to that for detergent‐purified supercomplexes (data from [24, 28]). The activities are shown at two different ionic strengths: 20 mm KCl (A) and 150 mm KCl (B). (C) Comparison of QH_2_ oxidation : O_2_ reduction activity of supercomplex in mitoplasts at 150 and 20 mm KCl, respectively. Error bars are ±SD of 3 independent measurements.

The supercomplex activity in mitoplasts, both at 20 and 150 mm KCl, is compared to that of supercomplexes purified in detergent (glyco‐diosgenin (GDN)), published previously [24, 28] (Fig. 3A,B). No activity was observed prior to cyt *c* addition. The data in Fig. 3B (150 mm KCl) show that in mitoplasts the activity increased with increasing cyt *c* concentration for up to ~ 50 nm cyt *c* and then remained at an essentially constant level of 15–20 e^−^/s up to ~ 1 μm cyt *c*. The activity increased slightly in the range 1 μm − 50 μm cyt *c*. Interestingly, in mitoplasts the supercomplex activities as a function of cyt *c* concentration were similar at 20 KCl and at 150 mm KCl (Fig. 3C), which contrasts the behavior observed previously for detergent‐solubilized supercomplexes [24]. We note that at 20 mm KCl, the supercomplex activity in mitoplast appears to be slightly higher at low cyt *c* concentrations than that in detergent‐purified supercomplex, up to 100 nm cyt *c* (Fig. 3A).

The catalytic activities of CIII_2_ and CIV, respectively, in mitoplasts were measured separately with 50 μm cyt *c*. The former was recorded by monitoring reduction of cyt *c* spectrophotometrically at 550 nm while CIV was inhibited by cyanide. The CIII_2_ activity in mitoplasts was 32 ± 1 e^−^/s and 42 ± 2 e^−^/s (mean ± SD, *n* = 3 independent measurements) at 20 and 150 mm KCl, respectively. These activities of CIII_2_ in mitoplasts are lower than that measured in supercomplexes isolated in GDN [24, 28] or for purified CIII_2_ [53] (80–90 e^−^/s), which is qualitatively consistent with data from earlier studies [54].

The activity of CIV was measured by monitoring the O_2_‐reduction rate in the presence of ascorbate and the electron mediator TMPD. The CIV activity in mitoplasts was 23 ± 5 e^−^/s or 35 ± 4 e^−^/s (mean ± SD, *n* = 3 independent measurements) at 150 or 20 mm KCl, respectively. As observed previously, the CIV activity in mitoplasts was lower than that measured in detergent‐purified supercomplex (450 e^−^/s (150 mm KCl) or 120 e^−^/s (20 mm KCl) [24, 28]).

The turnover rates outlined above are summarized in Table 1.

**Table 1:** Turnover rates in s^−1^ with 50 μm cyt *c*. Data with samples isolated in GDN are from [24, 28].

	Isolated in GDN		Mitoplasts	
	20 mm KCl	150 mm KCl	20 mm KCl	150 mm KCl
CIII_2_	80 ± 6	90 ± 20	32 ± 1	42 ± 2
CIV	120 ± 10	450 ± 20	35 ± 4	23 ± 5
SC	42 ± 1	60 ± 2	17 ± 8	15 ± 2

## Discussion

The activities of the detergent‐solubilized CIII_2_ and CIV are higher than those of these complexes in mitoplasts (Table 1). A recent study showed that detergent solubilization of CIV results in an increase in turnover activity by up to a factor of ~ 10 (depending on detergent used) [46]. This effect was attributed to detergent‐induced release of a membrane‐derived ligand from a steroid binding site near the K proton pathway of CIV [41, 42, 43, 44, 45]. When bound, the steroid ligand would slow the rate‐limiting proton uptake through this pathway, resulting in a lower activity compared to that with an empty steroid binding site [46].

For detergent‐solubilized supercomplexes, the QH_2_ oxidation : O_2_ reduction activity of the CIII_2_‐CIV supercomplex is rate limited by the cyt *c*‐mediated electron transfer from CIII_2_ to CIV [3, 24]. This rate‐limiting step is dependent on electrostatic interactions between cyt *c* and the supercomplex surface such that any changes in these interactions result in alteration of the supercomplex turnover activity. As mentioned above, data have further shown that within a CIII_2_‐CIV supercomplex, electron transfer from each CIII_2_ monomer to CIV occurs by 2D diffusion of the positively charged cyt *c* along the negatively charged supercomplex surface [17, 18, 24, 25, 26, 27]. Interactions between the negatively charged surface of the supercomplex and the positively charged cyt *c* are also manifested by an association of cyt *c* with supercomplexes upon isolation from, for example, *Ustilago maydis* [55] or *S. cerevisiae* [17, 18, 24, 25, 26, 27, 56]. Trouillard *et al*., [56] estimated that in intact *S. cerevisiae* mitochondria one cyt *c* molecule associates with each CIV. Because ~ 90% of CIV are found in CIII_2_CIV_1/2_ supercomplexes [52], approximately one cyt *c* would associate with each CIII‐CIV pair. Results from cryoEM studies have revealed that also in detergent‐purified supercomplexes, at 150 mm monovalent salt, a single cyt *c* associates with each CIII‐CIV pair where it is bound to either a CIII_2_ monomer, CIV or along intermediate positions [24]. At 20 mm monovalent salt, a substantial fraction of the supercomplex particles harbor two cyt *c* molecules per CIII monomer‐CIV pair [28].

The data in Fig. 3A,C show that at 20 mm KCl in mitoplasts, the supercomplex activity is 5–7 s^−1^ for cyt *c* concentrations of 10–20 nm, that is, up to a cyt *c* : supercomplex ratio of unity. Between 20 nm and 30 nm cyt *c*, the activity increases steeply to ~ 18 s^−1^. A similar behavior is observed for mitoplast at 150 mm KCl (Fig. 3B,C). Because the increase in activity occurs at an approximate cyt *c*:supercomplex ratio of unity, the data indicate that in this cyt *c* concentration range, each CIII monomer‐CIV pair recruits one cyt *c* at its cyt *c*‐binding surface. The data also indicate that at both low and high ionic strengths, the supercomplex‐cyt *c* interactions outcompete those of cyt *c* with the membrane surface at a cyt *c* : supercomplex ratio of unity. We also note that at 20 mm KCl the supercomplex activity in mitoplasts at cyt *c* concentrations 30–80 nm (cyt *c* : supercomplex ratios of 1–4) is higher than that in detergent solution at the same ionic strength (Fig. 3A). This observation indicates that the membrane facilitates cyt *c* binding at concentrations where cyt *c* binding is rate limiting for turnover of the supercomplex.

In contrast to the data with detergent‐solubilized supercomplexes, the supercomplex activity in mitoplasts remained at an approximately constant level in the range ~ 50 nm − 1 μm cyt *c*, that is, up to a cyt *c* : supercomplex ratio of ~ 50. This behavior can be explained in terms of a shift of the rate‐limiting step for the supercomplex activity in mitoplasts. While up to cyt *c* concentrations of ~ 30 nm (at 20 mm KCl) or ~ 80 nm (at 150 mm KCl) it is limited by cyt *c* binding and 2D diffusion along the supercomplex surface, at higher cyt *c* concentrations it is rate limited by the maximum catalytic activities of the supercomplex components.

We note that although the supercomplex activity at each cyt *c* concentration above ~ 50 nm cyt *c* is similar at 20 or 150 mm KCl, the general trend is that the activities are slightly higher at 20 mm than at 150 mm KCl (Fig. 3C), which would be due to slightly higher enzymatic turnover at the lower salt concentration. The slight turnover rate difference may be explained, for example, by a difference in the rate of the rate‐limiting proton uptake by CIV (see above, first paragraph in this section). The increase in activity above 1 μm cyt *c* (Fig. 3) is explained by 3D diffusion of cyt *c* molecules from the fraction of CIII_2_ that is not part of supercomplexes to any CIV in the membrane.

The activity at the lowest cyt *c* : supercomplex ratios, that is, up to ~ 50 nm cyt *c*, is presumably rate limited by cyt *c* binding and therefore expected to display an ionic strength dependence. The signal‐noise in the data is insufficient to model the cyt *c*‐concentration dependence, but we note that data from earlier studies in detergent solution revealed a larger Hill coefficient for cyt *c* binding to the *S. cerevisiae* supercomplex at 20 mm than at 150 mm KCl. This scenario was explained in terms of cooperative binding of more than one cyt *c* per supercomplex at low ionic strength [28]. The data in Fig. 3C qualitatively reveal a similar difference in the Hill coefficients, although it is insufficient to allow any quantitative evaluation. Nevertheless, we note that at 20 mm KCl, the supercomplex activity in mitoplasts at cyt *c* concentrations 30–80 nm, that is, a cyt *c* : supercomplex ratios of 1–4, is higher than that in detergent solution at the same ionic strength (Fig. 3A), and also higher than that in mitoplasts at 150 mm KCl (Fig. 3C). In other words, at physiological cyt *c*:supercomplex ratios (up to ~ 5, [3]), the activity at the lower ionic strength is the highest for all conditions used in this study, which is consistent with the notion that 20 mm KCl is an adequate mimic of the physiological electrostatic conditions [36].

To summarize, within the membrane environment, electrostatic interactions govern the supercomplex turnover rate until the cyt *c* : supercomplex stoichiometric ratio reaches unity. Beyond this point, the rate‐limiting step is determined by the catalytic constants of the supercomplex components CIII_2_ and CIV, rendering the overall turnover rate independent of ionic strength. This suggests that the functional discrepancies observed between detergent solutions and mitoplasts stem from inherent functional properties of the enzyme complexes rather than differences in electrostatic interactions.

## Author contributions

APL performed experiments, analyzed data, drafted paper, and prepared figures; MO and PB conceived and designed the project and wrote the final version of the manuscript.

## Data Availability

All data supporting the conclusions of this article are included within the article. No large‐scale datasets requiring public repository deposition were generated in this study.
